# Correlation of Three Dimensions of Palate with Maxillary Arch Form and Perimeter as Predictive Measures for Orthodontic and Orthognathic Surgery

**DOI:** 10.3390/children8060514

**Published:** 2021-06-17

**Authors:** Fadil A. Kareem, Aras Maruf Rauf, Tara Ali Rasheed, Falah Abdullah Hussain

**Affiliations:** 1 Pedodontics, Orthodontics and Preventive Dentistry Department, College of Dentistry, University of Sulaimani, Madam Mitterrand St., Sulaimani 46001, Iraq; fadil.kareem@univsul.edu.iq (F.A.K.); aras.rauf@univsul.edu.iq (A.M.R.); 2 Oral Surgery Department, College of Dentistry, University of Sulaimani, Madam Mitterrand St., Sulaimani 46001, Iraq; falah.hussein@univsul.edu.iq

**Keywords:** hard palate, three dimensions of the palate, Sulaimani City, arch form, arch perimeter

## Abstract

Hard palate is regarded as an important part of the human skull, which contributes to the separation of the oral and nasal cavities. The aims of the study were to investigate the morphology of the hard palate in order to create a general guideline of three-dimensional values of the palate in a Kurdish sample in the city of Sulaimani as well as determining the possible correlations between different palatal parameters in class I malocclusion with the maxillary arch form and perimeter. A retrospective study design was adopted by collecting 100 study models of orthodontic patients aged 16–24 years old attending different private dental clinics in the city of Sulaimani seeking orthodontic management. In this study, three-dimensional palatal measurements including depth, length, and width were measured in an attempt to discover their correlation with each maxillary arch form and perimeter. Additionally, measurements of inter-molar width, inter-canine width, and arch perimeter were carried out. About two-thirds of those seeking orthodontic treatment were females. Nearly 80% of the study sample had narrow palate followed by 15 and 5% of intermediate palate and broad palate, respectively. In regard to arch form, almost 90% of subjects were with tapered maxillary arch form and 10% of them with oval arch form. Males had increased dimensions compared to females, with significant differences, except in palatal depth in the molar area, and palatine height index, in which females showed increased dimensions than males but the differences were statistically non-significant. A strong positive correlation was observed between arch form and canine depth. In regard to arch perimeter, a strong negative correlation was found with molar depth and a medium positive correlation with each of canine depth, palatal width, and palatal length.

## 1. Introduction

The palate is situated in the maxillary region just below the maxillary sinus and the nasal cavity. It is composed of hard and soft palates [1]. The hard palate is regarded as an important part of the human skull, which contributes to the separation of the oral and nasal cavities [2]. It is also associated with dentition, especially maxillary teeth, and supports them [3]. Proper development of the soft and hard palate helps with proper phonation and other functional activities, along with proper development of the teeth [4].

Due to the morphology and position of the palate, it is considered to be a key anatomical structure that determines skeletal patterns. The palate might be affected by orthodontic treatment [5] because orthodontic treatment mainly requires modifications in arch dimensions for the correction of the existing malocclusions. Arch dimensions are often modified by the different archwires used during orthodontic treatment, which have an impact on the stability of the obtained results and stability of both maxillary and mandibular arch width, is stated to be affected by multiple factors prior to and after treatment [6]. Consequently, the dimensional changes ultimately affect arch form, reflecting the underlying bone morphology [7].

The palate is used as a reference for the replacement of missing teeth in prosthodontics. The assessment of palatal depth and arch form are very important factors during the election of artificial teeth. Moreover, the arch form acts as a crucial part of the oral cavity as it affects the selection of artificial teeth, and it is also related to the patient’s facial form. Depending on the individual’s facial form, their arch form can be easily identified. For instance, brachiocephalic persons typically have broad dental arches, whereas dolichocephalic persons have long or narrow dental arches and mesocephalic individuals usually have paraboloid or average dental arch form [8].

Sexual dimorphism is demonstrated by some palatal dimensions, so they can be used as predictors of sex. The sexual dimorphism of palatal dimensions is displayed in adults, and it is also demonstrated in children [9]. Additionally, sex probably plays an essential role in the determination of palatal dimensions and the changes that occur during developmental growth. The palatal dimensions are observed to be higher in males than in females [10]. On the other hand, in a study by Al-Mulla et al., who investigated the palatal depth of 50 maxillary study models of patients (18 males and 32 females) aged 15–20 years old, they reported that the difference between males and females was not significant [11].

The palatal height, length, and depth are influenced by various factors, including the size and shape of the jaws and the type of malocclusion. One of the goals of orthodontic treatment is the stability of the post-treatment outcomes, as the arch shape appears to return to the original shape [12].

Three-dimensional orthodontic calipers were used by Eid et al. to assess the width of the dental arch and the depth of the palatal vault, and no significant correlation was found between the perimeter of the arch and the depth of the palate [13]. In addition, knowledge of normal palatal dimension values can be used as a basis when studying oral developmental abnormalities [14]. Palatal dimensions have been reported to be influenced by ethnicity [15], dietary regimes [16], and environmental factors [10]. Every ethnic group and population affinity has its own unique facial and cranial form [17]. People may also have slightly different characteristics and facial shapes from individuals of other cultures in different countries. Furthermore, facial form is a part of the craniofacial complex, in which the palate morphology can be the major indicator of the anatomical structure in deforming the skeletal pattern [18].

The conduction of this study aimed at the investigation of hard palate morphology, creating general guidelines of three-dimensional values of the palate in a Sulaimani Kurdish sample identifying gender differences (if present). Moreover, finding out possible associations and correlations between the different palatal parameters in normal class I malocclusion with the maxillary arch form and perimeter was another purpose of the study conducted.

## 2. Material and Methods:

The ethical committee, College of Dentistry/University of Sulaimani agreed to give approval to perform the present study with an ethical number (23/21). The sample comprised 100 study models of orthodontic patients aged 16–24 years old attending different private dental clinics in the city of Sulaimani seeking orthodontic management. A retrospective study design was adopted during the study.

Criteria of sample selection:

1. Mild to moderate Class I malocclusion cases with a normal upper midline.
2. Complete set of permanent dentition except for the third molar.
3. No history of previous orthodontic and orthognathic treatments.
4. No congenital and developmental abnormalities.
5. No history of extracted permanent teeth.
6. No history of significant respiratory and allergic problems.

## 3. Measurements:

**Figure 1:**
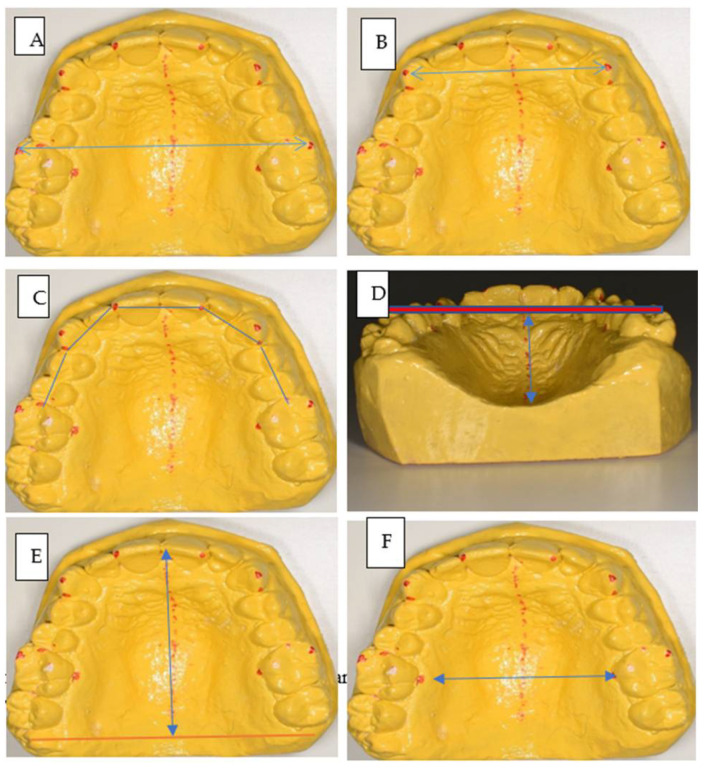
Measurements, (**A**) inter-molar width, (**B**) inter-canine width, (**C**) arch perimeter, (**D**) palatal depth, (**E**) palatal length, (**F**) palatal width.

Linear measurements: As illustrated in Figure 1, these include:

1. Maxillary arch measurements, which were performed via digital Vernier with an accuracy of 0.01 (Mitutoyo, Japan).
  - Inter-molar width: the linear distance at the level of the molar mesio-buccal cusp tips [7]: Figure 1A.
  - Inter-canine width: the linear distance at the level of the canine cusp tips [19] Figure 1B.
  - Arch perimeter: the dental arch perimeter was obtained by summation of five segmental measurements: from the mesial aspect of the first molar to the distal aspect of cuspids, from the distal aspect of the cuspids to the distal aspect of central incisors on both left and right sides, and from the distal aspect of the right central incisors to the distal aspect of the left central incisors [19] (Figure 1C).
2. Palatal measurements: performed using the digital Vernier.
  - Palatal depth: the vertical distance at the mid-palatal suture measured the level of the first molars and canines by adjusting a metal ruler on the occlusal surface of the first molars when the ruler touches the mesiobuccal cusp tip as the molar depth (MD) (Figure 1D) and measuring the distance from the horizontal plane touching the tips of the right and left canines and the mid-palatal suture as the canine depth (CD)
  - Palatal length: this was measured from the anterior part of the palate, which is from the linear contact point of the maxillary central incisors, (a), to the posterior part of the palate which is the most distal point of the maxillary permanent molars (b) [8] (Figure 1E).
  - Palatal width: the palatal width was measured from the maxillary first molars of one arch to the opposite arch, at the level of the edge of the palatal gingival sulcus [20] (Figure 1F).

Indices:

1. 1. Palatine Height Index (PHI): In order to assess the height of the palate at the molar, The calculation was performed using the PHI formula below:
  $$\mathrm{Index}\;\mathrm{of}\;\mathrm{Palatine}\;\mathrm{Height}\;\mathrm{Formula}=\frac{\mathrm{Palatal}\;\mathrm{Height}}{\mathrm{Palatal}\;\mathrm{Width}}\times 100$$

On the basis of the above formula and according to the study performed by Maria CM, the palatal depth was categorized into three types, which were as follows [1]:

- Low palate: if the values were ≤27.9%.
- Medium palate: if the values ranged between 28.0 and 39.9%.
- High palate: if the values were greater than 40.0%.

2. Arch Form Index (AFI): the arch form for all study casts was determined by measuring each of the inter-canine width (ICW), CD, inter-molar width (IMW), and molar depth (MD). Based on these observations, the arch form ratio was calculated depending on the AFI formula as illustrated in the following equation.
  $$\mathrm{Arch}\;\mathrm{Form}\;\mathrm{Index}\;\mathrm{Formula}=\frac{\mathrm{CD}}{\mathrm{ICW}}\times \frac{\mathrm{IMW}}{\mathrm{MD}}$$

Each cast was then classified into three categories, namely square, ovoid, and tapered, derived from their ratio as explained below. Arch form ratio was obtained in accordance to the study by Budiman in [21]:

- Arch form ratio is <45.30%, which means square arch form.
- Arch form ratio is between 45.30 and 53.37%, which means oval arch form.
- Arch form ratio is more than 53.37%, which means tapered arch form.

3. Palatine Index (PI): it was calculated through the use of the specific formula adopted by Khatiwada et al. [22].
  $$\mathrm{Palatine}\;\mathrm{Index}\;\mathrm{Formula}=\frac{\mathrm{Palatine}\;\mathrm{width}}{\mathrm{Palatine}\;\mathrm{length}}\times 100$$

PI means the ratio of the palatine width to the palatine length which is expressed as a percentage.

(1) If PI is less than 79, the palate is narrow (Leptostaphyline).
(2) If PI is between 80 and 84.9, the hard palate is intermediate in width (Mesostaphyline).
(3) If PI is 85 or more, the hard palate is broad (Brachystaphyline).

## 4. Statistical Analysis

Data entry was carried out with the 25th version SPSS computer program out. Normality test was performed using the Shapiro–Wilk test. Descriptive and inferential statistics were calculated.

## 5. Results

Descriptive analysis of various palatal dimensions including depth at molar and canine areas, palatal width and palatal length in addition to IMW, ICW, and arch perimeter are all exhibited in Table 1.

Most participants were females, comprising 67% of the study sample as shown in Table 2. Frequency and percentages of arch form, PHI, and PI were calculated in Table 2. Furthermore, the PHI of the study sample was assessed. All of the subjects had a high type of palate. The results of PI demonstrated that 80% of the study sample had a narrow palate followed by 15% and 5% of the intermediate palate and broad palate, respectively. In regard to arch form, almost 90% of subjects had tapered maxillary arch form and 10% of them with oval arch form.

Gender differences among different variables are exhibited in Table 3. It was observed that in most the dimensions, males had increased dimensions compared to females with significant differences except in the palatal depth at the molar area and PHI, in which females showed an increase in the dimensions than males but the differences were statistically non-significant.

A strong positive correlation was observed between arch form and CD with a medium positive correlation between arch form and PHI. In regard to the arch perimeter, a strong negative correlation was found with MD and a medium positive correlation with each of CD, palatal width, and palatal length (Table 4). Results of the multiple regression analysis reveal that all dimensions of the palate were significantly associated with arch perimeter as shown in Table 5, whereas only MD and CD were significantly associated with the arch form (Table 6).

## 6. Discussion

Different populations and ethnic groups exhibit variable dental arch dimensions and characteristics [23], encouraging researchers worldwide to document dental arch dimensions and forms in several populations and races. Although the establishment of normative value needs a larger sample size and effort, the study on 100 patients’ casts might provide a general guideline of dimensions of arch, palate, and form of the dental arch [24]. Exact assessment of the hard palate provides many clinical considerations, notably in various disciplines such as orthodontics, orthognathic surgeries, palatal implants, cleft palate management, and treatment of obstructive sleep apnea [2,25]. However, besides finding out the general guidelines of palatal and arch dimensions, determining the existence of any association and correlation of palatal dimensions and perimeter as well as arch form were another aim of the current study.

The study depends on the important aspect of establishing a set of norms on various arches, and palatal parameters present significant clinical considerations in various disciplines in dentistry. The arch form norm of the population probably facilitates the selection of arch wires during orthodontic treatment courses, for instance.

An increase in dental arch dimensions occurs at up to 9 years in the incisor region, whereas it may reach 13 years of age in the other regions of the dental arch. Then, after that age, little change occurs [26]. This is why the age range of the present sample study included 16–24-year-old orthodontic patients.

Study casts were selected as a raw material for conduction of the study as it can offer much information about the intended case, and it has also many advantages, such as determination of space available and required calculation, arch widths, lengths, and perimeters, with the aid of digital Vernier as analyzing software [27].

All of the subjects who participated in the current study had a high type of palate. The results of PI demonstrated that 80% of the study sample had narrow palate followed by 15 and 5% of the intermediate palate and broad palate, respectively. In regard to arch form, almost 90% of subjects had a tapered maxillary arch form, and 10% of them had an oval arch form, with zero cases with square arch form.

1. Palatal width: the mean of the palatal width was 34.05 ± 3.11 mm in our study; Khatiwada et al. in 2020 did a study on a Nepalese population and stated that the mean palatal width was 40.63 ± 3.76 mm [22]. Mustafa et al., in a similar study on Jordanians, found it to be 45.05 ± 2.47 mm in males and 40.23 ± 2.01 mm in females. Similarly, Annapurna et al. showed that the average palatal width was 38.2 ± 03.2 mm in 60 patients in India attending a government hospital [28]. Accordingly, the mean palatal width of the current study sample was less than all the studies done before.

Significant gender differences were reported in the palatal width of the present study, which was in agreement with that performed on the Iraqi population by Ahmed et al. [8]. Similarly, gender differences in palatal width were also noted on Yemeni and Jordanian samples by Al-Zubair et al. and Mustafa et al. [29,30]. Conversely, Klosek et al. stated no gender difference in palatal width nor in palatal height [31].

2. Palatal length: the mean palatal length of the present study was 46.11 ± 3.40 mm. It was found to be 41.58 ± 3.48 mm in a Nepalese population by Khatiwada et al. [22]. Mustafa et al., who examined 150 dental casts of adult persons found the mean palatal length to be 43.91 ± 2.65 and 39.53 ± 2.73 mm, respectively, and reported significant gender differences [30]. Moreover, it was 51.4 ± 5.8 mm, 51.65 ± 4.7 mm, 50.82 ± 03.59 mm, 43.54 ± 0.28 mm, and 49.74 mm in studies carried out by Klosek et al., Shalaby et al., Dave et al., and Jotania et al. respectively [31,32,33,34].

Significant gender dimorphism was observed in our study with increased dimension for males, whereas Ahmed et al. [8] found no significant sex differences in respect to palatal length which were in controversy with the findings of Khatiwada et al. in 2020 and Al-Zubair et al., who reported longer palates in females when compared to males [22,29].

3. Palatal depth: the mean palatal depth in the molar area was 22.53 ± 2.10 mm in our sample. It was 14.90 ± 2.05 mm and 20.76 ± 3.1 mm by Khatiwada et al. [22] and Alshahrani et al. [35], respectively. Alkadhi et al. in 2018 on Saudi adults noted a mean palatal height of 20.90 ± 2.08 in males and 20.54 ± 2.09 mm in females [36]. Nahidh et al. in 2012 measured, on an Iraqi sample, a mean palatal height in dental casts with type 1 malocclusion and found it to be 14.9 ± 1.78 mm [37].

Furthermore, Sarilita et al., in a study of the morphology of hard palate on dry skulls [38] reported the mean height as 11.54 ± 2.4 mm, while Klosek et al., Dave et al., and Tomaszewska et al. found it to be 17.7 ± 4.2 mm, 9.87 ± 0.23 mm, and 13.1 ± 2.7 mm, respectively [31,33,39]. Another study on the Kenyan African skull by Hassanali et al. palatal height was 12.2 ± 01.6 mm [40]. Accordingly, the mean palatal depth of the present study was more than all previously mentioned studies from different parts of the world identifying deeper palate in a Kurdish sample.

No significant gender difference was reported in palatal height in the molar area in the present study. Similar results were acquired on an Iranian population [41]. Furthermore, Tsai and Tan demonstrated no gender differences [42]. On the contrary, Al-Zubair [29] and Thalider [14] showed that palatal height in the molar site was greater in females than in males. This contradiction could be attributed to ethnic differences among studied populations.

Generally, differences in palatal measurements have been encountered in the literature among individuals with respect to growth pattern of facial region, environmental and genetic background, and pathologies such as enlarged tonsils, nasal allergies, or prolonged mouth breathing [22]. Although controversies were reported by different investigators about gender differences of palatal measurements [29,43,44], the findings of the current study displayed higher values in all measured dimensions in males compared to females except palatal depth at molar area and PHI.

4. Arch form: in regard to arch form, the tapered maxillary arch form was the most frequent type, followed by an oval arch form, while the squared shape maxillary arch was not observed in the current study. The oval maxillary arch form dominates in the Sudanese population [24]. A recent Saudi study found the most prevalent arch form to be the narrow tapered followed by the narrow ovoid [45].

The tapered arch shape was the most prevalent maxillary arch form in both the Indian and the Malaysian population. In an Indian sample, the majority of them had tapered arch form, and the remaining had ovoid arch form, without any squared maxillary arch form. While in the Malaysian population, although the majority of them had tapered arch form, there were only some with oval maxillary arches and few with squared arch form [4]. In another study on the Malaysian population by Othman et al., the ovoid maxillary arch form was the greatest, followed by tapered and then square [20]. It was stated that Indian people tend to have a narrow arch form in a study performed by Sahoo et al. [46].

5. Arch Perimeter: With reference to what was stated in the results, all dimensions of the palate were significantly associated with the arch perimeter. On the other hand, a strong correlation of arch perimeter was reported with MD and a medium correlation with CD, palatal width, and palatal length. On the contrary, no significant correlation between the arch perimeter and the depth of the palate and a significant correlation of the arch perimeter with palatal width was found in a study done by Eid et al. [13] on Egyptians. Furthermore, in another study on the Iraqi population by Salman in 2001 [47], no correlation was found between arch perimeter and palatal depth. At the same time, a moderate correlation of maxillary perimeter with palatal width was demonstrated by Salman in 2001 which was in concordance with the present study. Different ethnic groups, sample size, different landmarks, and measurement devices with a different age groups of the study sample may be behind these controversies.

Expansion of the present study is recommended and suggested by the authors to a larger study to set norms for palatal and arch dimensions to reflect the characteristics of the Iraqi Kurdish population.

## 7. Limitations

Further studies with larger sample sizes are mandatory and essential in order to better reflect the characteristics of the Iraqi Kurdish population as a whole. Furthermore, selecting either adolescents or young adults separately gives a more accurate representation of the measurements due to the prolonged growth, especially in male teenagers.

## 8. Conclusions

Morphometric knowledge of the hard palate surely has beneficial effects on various disciplines of dentistry, encompassing orthodontic and orthognathic treatments, for instance. The majority of Kurdish individuals in the city of Sulaimani possess a high narrow palate and tapered arch form. Males have larger dimensions than females with significant differences, except in palatal depth in the molar area and palatine height index, in which females had non-statistically significant increased dimensions than males. A strong positive correlation was observed between arch form and canine depth. In regard to arch perimeter, a strong negative correlation was found with molar depth and a medium positive correlation with each of canine depth, palatal width, and palatal length.

## Figures and Tables

**Table 1:** Descriptive statistics of different variables.

	Variables	No.	Min.	Max.	Mean ± SD
1.	Inter-molar width	100	44.00	56.00	50.32 ± 2.86
2.	Inter-canine width	100	29.50	38.50	34.63 ± 2.35
3.	Palatal depth at molar area	100	19.00	28.00	22.53 ± 2.10
4.	Palatal depth at canine area	100	8.00	13.50	11.25 ± m 1.73
5.	Palatal length	100	40.00	52.00	46.11 ± 3.40
6.	Palatal width	100	28.20	43.05	34.05 ± 3.11
7.	Arch perimeter	100	66.00	86.00	77.89 ± 5.77
8.	Palatine Height Index	100	50.00	84.85	66.69 ± 8.25
9.	Palatine Index	100	62.50	94.15	74.00 ± 6.33
10.	Arch form	100	48.00	96.00	73.25 ± 13.38

**Table 2:** Frequency and percentages of different variables of the study sample.

Variables		Frequency and Percentages
Gender	Males	33
	Females	67
Arch form	Square (<45.30%)	
	Oval (between 45.30–53.37%)	10
	Tapered (more than 53.37%)	90
Palatine Height Index	Low palate (≤27.9%)	−
	Medium palate between 28.0 and 39.9%	−
	High palate (more than 40.0%)	100
Palatine Index	Narrow palate (≤79)	80
	Intermediate palate (80–84.9)	15
	Broad palate (85 or more)	5

**Table 3:** Gender differences of different variables of the study sample.

	Variables	Gender	No.	Mean ± SD	t-Value	*p* Value
1.	Inter-molar width	Males	33	53.32 ± 1.02203	10.815	0.000
		Females	67	48.85 ± 2.26		
2.	Inter-canine width	Males	33	36.89 ± 1.16	9.007	0.000
		Females	67	33.53 ± 1.97		
3.	Palatal depth at molar area	Males	33	22.09 ± 1.85	1.500	0.137
		Females	67	22.75 ± 2.19		
4.	Palatal depth at canine area	Males	33	12.57 ± 0.501	6.290	0.000
		Females	67	10.60 ± 1.764		
5.	Palatal length	Males	33	49.1515 ± 1.09320	8.009	0.000
		Females	67	44.61 ± 3.153		
6.	Palatal width	Males	33	36.87 ± 1.00	8.228	0.000
		Females	67	32.6 ± 2.85		
7.	Arch perimeter	Males	33	85.04 ± 1.16	17.693	0.000
		Females	67	74.37 ± 3.35		
8.	Palatine Height Index	Males	33	60.02 ± 6.13	6.861	0.000
		Females	67	69.97 ± 7.12		
9.	Palatine Index	Males	33	75.07 ± 2.79	1.185	0.239
		Females	67	73.47 ± 7.44		
10.	Arch form	Males	33	0.829 ± 0.082	5.866	0.000
		Females	67	0.684 ± 0.128		

**Table 4:** Correlation of three dimensions of the palate with arch form and arch perimeter.

Palatal 3D Measurements		Correlation Coefficient (r)	
		Arch Form	Arch Perimeter
1.	Palatal molar depth	−0.36	−0.90
2.	Palatal canine depth	0.83	0.53
3.	Palatal width	0.46	0.58
4.	Palatal length	0.16	0.56
5.	Palatine Hight Index	−0.58	−0.47
6.	Palatine Index	0.33	0.12

**Table 5:** Multiple regression analysis of arch perimeter (as a dependent variable) and several co-variates (*n* = 4).

		Unstandardized Coefficients		Standardized Coefficients	*t*-Value	*p* Value
		B	SE	Beta		
1.	Palatal molar depth	−1.05	0.216	−0.382	−4.86	0.000
2.	palatal canine depth	0.673	0.273	0.203	2.46	0.015
3.	Palatal width	0.398	0.158	0.215	2.52	0.013
4.	Palatal length	0.934	0.158	0.551	5.9	0.000

**Table 6:** Multiple regression analysis of arch form (as a dependent variable) and several co-variates (*n* = 4).

		Unstandardized Coefficients		Standardized Coefficients	*t*-Value	*p* Value
		B	SE	Beta		
1.	Palatal molar depth	−0.028	0.002	−0.435	−12.24	0.000
2.	palatal canine depth	0.069	0.003	0.9	24.24	0.000
3.	Palatal width	0.001	0.002	0.025	0.645	0.521
4.	Palatal length	−0.002	0.002	−0.62	−1.47	0.145

## Data Availability

The data preserved by the authors and available on request throgh this email: aras.rauf@univsul.edu.iq.

## Abbreviations

Molar Depth	MD
Canine Depth	CD
Palatine Height Index	PHI
Arch Form Index	AFI
Inter-Canine Width	ICW
Inter-Molar Width	IMW
Palatine Index	PI
